# Effects of Air Pressure and Holding Time on Muscle Blood Flow Promotion in Pneumatic Squeeze Hold

**DOI:** 10.7759/cureus.71584

**Published:** 2024-10-16

**Authors:** Masaaki Nakajima, Tomoka Tsuro, Akemi Endo

**Affiliations:** 1 Department of Physical Therapy, School of Health Science and Social Welfare, Kibi International University, Takahashi, JPN; 2 Physical Medicine and Rehabilitation, Medical Concierge Co. Ltd., Okayama, JPN

**Keywords:** carbon dioxide (co2), dormant capillary network, near-infrared spectrometry, precapillary sphincter muscle, sto2

## Abstract

Introduction

This study aimed to investigate the effects of air pressure and holding time on the promotion of muscle blood flow using a squeeze-hold technique with a pneumatic cuff in healthy college students.

Methods

Subjects (n = 12) were placed in the prone position, and a pneumatic cuff was secured around the left lower leg. The cuff was inflated to predetermined pressures (50, 100, and 200 mmHg) to squeeze the lower leg muscles. After the pressure was held for predetermined time periods (20 seconds, one minute, three minutes, five minutes, and seven minutes), the cuff was deflated to release the associated compression in the lower leg muscles. During the intervention and after release, the hemodynamics of the left gastrocnemius muscle were measured using a muscle infrared spectrometer.

Results

At 50 mmHg, the promotion of muscle blood flow was absent at all holding times. At 100 mmHg, the effect of enhanced muscle blood flow was observed as follows: for up to one minute after release with holding times of 10 seconds and 1 minute, up to two minutes after release with a holding time of three minutes, and up to eight minutes after holding times of five and seven minutes. At 200 mmHg, a muscle blood flow-promoting effect was observed for up to one minute after release with a holding time of 10 seconds, up to two minutes after release with a holding time of one minute, and up to 10 minutes after release with holding times of three, five, and seven minutes.

Conclusion

The analysis indicated that a cuff pressure of at least 100 mmHg was necessary to achieve a significant blood flow-promoting effect. Furthermore, longer holding times at higher pressures produced more sustained increases in blood flow. These findings suggest that the squeeze-hold technique, with appropriate pressure and duration, can effectively increase muscle blood flow.

## Introduction

The squeeze-hold technique to promote muscle blood flow, which is squeezing the muscle with a pneumatic cuff and holding it in that state for a certain period, can promote blood flow after release [1]. Two mechanisms of action are involved in promoting blood flow. One is the release of nitric oxide (NO) due to shear stress on vascular endothelial cells upon restoration of blood flow after pressure release. Shared stress from blood flow to vascular endothelial cells causes NO to be released from vascular endothelial cells, which relaxes vascular smooth muscle and promotes blood flow [2-5]. The second mechanism is an increase in carbon dioxide concentration due to the metabolism of muscle cells during blood flow restriction. Cuff compression restricts blood flow. By maintaining this state, the concentration of carbon dioxide in the muscle tissue increases owing to the metabolism of muscle cells. The decrease in muscle volume (decrease in volume per cell: increase in cell density) caused by squeezing accelerated the rate of increase in carbon dioxide concentration. The decrease in pH due to an increase in carbon dioxide concentration relaxes the vascular smooth muscle [6], thus promoting blood flow after release.

Effects on the dormant capillary network are as follows: Blood flow to the capillary network is controlled by the precapillary sphincter muscle (vascular smooth muscle), which in turn is controlled by the sympathetic vasoconstrictor nerve. In addition, a network of dormant capillaries is present. Blood flow to the active capillary network is enhanced by heat or massage; however, blood flow to the dormant capillary network is not expected to be enhanced. By contrast, squeeze-hold relaxes all precapillary sphincter muscles in the target area; therefore, it has an effect even on dormant capillary networks and thus can be expected to have an excellent flush-out effect on metabolites and inflammatory substances.

Previous studies of squeeze hold have evaluated muscle blood flow under only one condition: cuff pressure of 200 mmHg and hold time of 10 minutes [1]. For the clinical application of squeeze hold, the blood flow-promoting effect of squeeze hold is expected to vary depending on the strength of the shear stress caused by blood flow when the squeeze hold is released and the degree of decrease in tissue pH. Therefore, to apply squeeze hold in clinical practice, understanding the blood flow-promoting effect of different cuff pressures and holding times is necessary to verify the clinical effect.

The purpose of this study was to clarify the effects of air pressure and holding time on the promotion of muscle blood flow during the squeeze-hold technique using a pneumatic cuff in healthy subjects.

## Materials and methods

**Figure 1:**
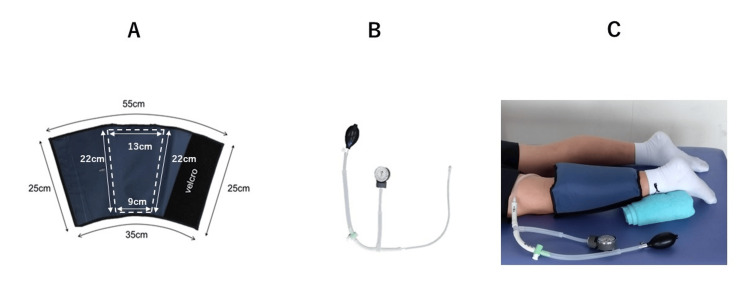
Pneumatic cuff The pneumatic cuff was made of sturdy nylon and was sized to cover the lower leg. An internal air chamber is present in it, which applies pressure on the gastrocnemius muscle. Velcro makes it easy to put on and take off. The valve on the surface of the cuff enables the insertion of air (A). A pressure gauge is integrated into the pump's design, which allows for inflating the air to a specified pressure (B). A pneumatic cuff was wrapped around the subject's left lower leg as they assumed a prone position (C).

A cuff with a pneumatic chamber was designed and attached to the back of the lower leg using an easily detachable Velcro strap (Figure 1).

When air is pressurized into the pneumatic chamber, it expands inward and compresses the gastrocnemius muscle. Settings of the air pressure value of the pneumatic cuff and the pressure holding time were based on a previous study using a pneumatic cuff [1] in which a 200 mmHg pressure and 10-minute hold-time were found to promote muscle blood flow for at least 10 minutes after release. Functional anoxia was achieved after seven minutes of pressure holding. Moreover, considering that the expansion pressure of intermittent pneumatic compression therapy for the prevention of edema and deep vein thrombosis is approximately 50 mmHg in the lower limbs, the pressure was set to three values, i.e., 50, 100, and 200 mmHg, and the holding time was set to five limits, i.e., 20 seconds and one, three, five, and seven minutes.

Evaluation of muscle blood flow

Near-infrared spectrometry is used as a non-invasive method to measure changes in blood circulation in the muscles and was used in this study to evaluate muscle blood flow. Muscle tissue hemodynamics (StO_2_, total Hb), which can be measured by near-infrared spectroscopy, reflects the balance between oxygen supply and consumption in muscle tissue [7] and can capture changes in muscle blood flow at the local muscle tissue level [8-10].

Subjects

This study included 12 healthy college students from Kibi International University (Table 1). The average age of the subjects was 20.3 ± 0.5 years. The average BMI was 22.2 ± 0.8. This experiment was approved by the Kibi International University Experimental Ethics Committee (approval no. 21-44). Each subject provided informed consent to participate in the study according to the procedures of the institutional review board. After obtaining consent, each participant was asked to complete a medical history questionnaire. Participants were excluded if they had a history of back or lower limb problems.

**Table 1:** Demographic information of the subjects

	Age (y)	Sex	Height (cm)	Weight (kg)	BMI
Subject 1	20	male	187	78	22.3
Subject 2	20	male	176	65	21.0
Subject 3	20	male	164	61	22.7
Subject 4	21	male	170	61	21.1
Subject 5	20	male	170	65	22.5
Subject 6	21	male	170	67	23.2
Subject 7	20	male	168	65	23.0
Subject 8	21	male	170	65	22.5
Subject 9	20	male	170	67	23.2
Subject 10	21	male	178	70	22.1
Subject 11	20	male	180	68	21.0
Subject 12	20	male	180	70	21.6
Mean	20.3				22.2
SD	0.5				0.8

Experimental protocol

**Figure 2:**
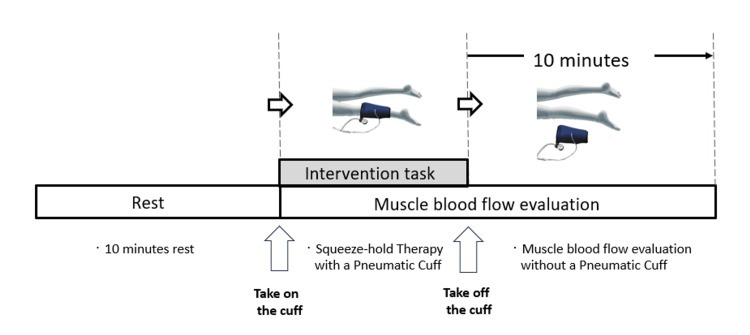
Experimental protocol After a 10-minute rest period, the subject was placed in a supine position on a bed with a near-infrared spectrometer probe attached onto the left gastrocnemius muscle and a pneumatic cuff wrapped around the left lower leg. The cuff was pressurized and held under predetermined conditions. The pneumatic cuff was decompressed and removed after the prescribed time. Muscle hemodynamics were measured from the start of the intervention until 10 minutes after the end of the intervention.

After 10 minutes of rest, the subject was placed in the prone position, the probe of the near-infrared spectrometer was attached to the upper surface of the gastrocnemius muscle on the posterior aspect of the left lower leg, and a cuff was wrapped around the lower leg (Figure 2).

The cuff was inflated to predetermined pressures (50, 100, and 200 mmHg) for five seconds to restrict the blood flow to the lower leg. After inflating the cuff for predetermined time periods (20 seconds, one minute, three minutes, five minutes, and seven minutes), it was deflated to release the restricted blood flow in the leg, and the patient was allowed to rest for 10 minutes. During the intervention, the hemodynamics of the left gastrocnemius muscle were measured using a muscle infrared spectrometer (BOM-L1 TR, OmegaWave, Inc., Tokyo, Japan), and the data were stored on a computer using an A/D converter (PowerLab; AD Instruments, Sydney, Australia).

Statistical processing was performed using a one-way analysis of variance with repetition for StO_2_ values at rest and every minute for 10 minutes after the release of blood flow restriction and Dunnett’s test for multiple comparisons. The significance level for all statistical analyses was set at 5%. Stat View (version 5.0, SAS Institute Inc., USA) was used for statistical analysis.

## Results

At 50 mmHg, StO_2_ at the time point just before release was significantly lower than that at rest at all holding times. The promotion of muscle blood flow was absent at all holding times.

At 100 mmHg, at the time point just before release, StO_2_ was significantly lower than that at rest for all holding times. The effect of promoting muscle blood flow was observed up to one minute after release for holding times of 10 seconds and one minute, up to two minutes after release for a holding time of three minutes, and up to eight minutes after release for holding times of five and seven minutes.

At 200 mmHg, at the time point just before release, StO_2_ was significantly lower than that at rest at all holding times. The muscle blood flow-promoting effect was observed for up to one minute after release with a holding time of 10 seconds, up to two minutes after release with a holding time of one minute, and up to 10 minutes after release with holding times of three, five, and seven minutes. Because a large difference in the duration of the blood flow promotion effect between holding times of one and three minutes was observed, additional experiments were conducted for a holding time of two minutes, where StO_2_ at the time point just before release was found to be significantly lower than that at rest, and the blood flow promotion effect was observed for up to four minutes after release (Table 2).

**Table 2:** Change in skeletal muscle oxygen saturation At an air pressure of 50 mmHg, no effect of increasing StO_2_ was observed. When the air pressure was 100 mmHg and 200 mmHg, the higher the air pressure and the longer the holding time, the higher the effect of StO_2_ increase. Mean ± standard deviation, * p ＜ 0.05 vs. at rest (paired t-test), ψ  p ＜ 0.05 vs. at rest (Dunnett)

		Elapsed time from the release																							
Air pressure value	Pressure holding time	At rest		Just before the release		One minute		Two minutes		Three minutes		Four minutes		Five minutes		Six minutes		Seven minutes		Eight minutes		Nine minutes		Ten minutes	
50 mmHg	20 sec hold	71.2±4.3		68.4±4.7	*	72.4±4.6		72.2±4.2		72.1±4.2		72.1±4.1		71.9±3.5		72.0±4.7		71.8±5.0		72.4±4.1		72.6±3.7		72.8±4.0	
	1 min hold	68.4±4.2		63.9±0.6	*	70.7±4.3		68.8±5.7		68.6±5.1		68.7±4.6		68.7±4.0		68.7±4.7		69.0±4.0		69.2±4.5		69.1±4.5		69.8±4.0	
	3 min hold	66.9±4.9		63.9±4.5	*	69.7±6.1		68.6±5.5		68.2±4.5		68.7±3.2		67.8±4.4		67.8±4.7		68.4±4.4		68.4±4.4		68.2±5.3		68.4±4.0	
	5 min hold	70.1±3.0		66.6±3.6	*	70.9±2.9		70.3±3.3		70.9±3.0		71.1±2.9		70.7±3.0		71.4±3.1		71.2±2.9		70.8±2.7		71.1±2.8		70.8±2.7	
	7 min hold	70.5±5.3		67.9±3.2	*	71.1±5.5		71.4±6.2		71.3±6.1		71.5±6.3		71.9±6.1		71.5±5.9		71.9±6.1		71.4±5.9		72.4±5.0		72.2±4.9	
100 mmHg	20 sec hold	68.8±1.8		66.5±2.2	*	71.1±3.2	ψ	69.8±2.8		70.1±2.2		70.0±2.3		70.2±2.8		70.0±2.7		70.1±2.8		70.1±2.7		70.1±2.3		70.2±2.0	
	1 min hold	70.1±2.6		58.9±4.9	*	76.1±1.8	ψ	72.0±3.0		70.9±3.5		71.0±2.8		71.2±3.3		71.3±3.9		71.2±3.8		71.0±4.3		70.4±3.0		70.9±2.9	
	3 min hold	68.0±3.4		39.1±8.7	*	77.3±3.6	ψ	72.8±3.3	ψ	70.1±3.1		69.3±4.5		68.1±5.3		68.9±3.5		68.7±3.9		68.8±3.7		68.7±3.0		68.8±3.6	
	5 min hold	73.0±3.0		37.7±6.6	*	79.1±2.8	ψ	77.9±3.5	ψ	76.7±4.7	ψ	75.6±3.3	ψ	75.5±3.3	ψ	75.6±3.5	ψ	75.9±3.1	ψ	74.8±3.1	ψ	75.1±4.0		75.0±4.3	
	7 min hold	70.9±5.3		35.4±4.1	*	77.1±1.3	ψ	76.0±1.8	ψ	75.0±1.7	ψ	75.1±1.7	ψ	75.2±1.9	ψ	74.9±2.5	ψ	75.2±2.1	ψ	74.5±2.0	ψ	74.3±2.3		74.2±2.6	
200 mmHg	20 sec hold	66.9±4.0		63.6±3.8	*	69.4±3.6	ψ	67.1±5.2		67.2±5.0		67.3±4.1		67.1±5.1		67.2±5.0		67.3±4.4		67.1±3.7		67.0±3.9		66.8±4.5	
	1 min hold	66.9±1.4		54.8±3.4	*	71.6±2.0	ψ	69.6±1.6	ψ	68.8±1.0		68.1±0.8		68.3±0.9		68.6±1.6		68.6±1.1		68.4±1.9		68.9±1.9		68.7±1.2	
	3 min hold	67.8±2.4		38.8±10.0	*	76.3±2.1	ψ	73.7±2.3	ψ	71.4±3.1	ψ	71.4±3.2	ψ	71.4±3.1	ψ	70.9±2.6	ψ	70.7±2.8	ψ	71.0±2.7	ψ	70.6±3.0	ψ	70.5±2.6	ψ
	5 min hold	65.6±2.3		31.8±6.6	*	74.0±0.9	ψ	73.3±1.6	ψ	71.0±2.1	ψ	70.1±2.5	ψ	70.1±2.3	ψ	69.8±2.6	ψ	69.5±2.5	ψ	69.5±2.3	ψ	69.7±2.0	ψ	70.2±1.5	ψ
	7 min hold	65.4±2.5		30.7±7.1	*	73.9±0.6	ψ	74.5±1.7	ψ	71.4±1.4	ψ	69.8±2.8	ψ	70.4±1.8	ψ	69.5±2.8	ψ	69.5±3.1	ψ	69.7±3.2	ψ	69.2±2.4	ψ	69.7±2.7	ψ

Regarding the total Hb, at 50 mmHg, no change was observed in the value of total Hb at the time point just before release and over time for all holding times compared with that in the resting state.

At 100 mmHg, the value of the total Hb for the seven-minute holding time was greater than that at one and two minutes after release.

At 200 mmHg, the values of the total Hb for holding times of three, five, and seven minutes were large from one to 10 minutes after release (Table 3).

**Table 3:** Change in the total Hb (total blood volume) At an air pressure of 50 mmHg, there was no change in the total Hb. A high value of total Hb was observed at two minutes after release when air pressure was 100 mmHg and holding time was seven minutes. At 200 mmHg air pressure, high total Hb values were observed for more than 10 minutes after opening under the condition that the holding time was more than three minutes. The air pressure of 200 mmHg seems to be appropriate to obtain the increase in the total Hb. Mean ± standard deviation, ψ  p ＜0.05 vs. at rest (Dunnett)

		Elapsed time from the release																							
Air pressure value	Pressure holding time	At rest		Just before the release		One minute		Two minutes		Three minutes		Four minutes		Five minutes		Six minutes		Seven minutes		Eight minutes		Nine minutes		Ten minutes	
50 mmHg	20 sec hold	1.06±0.12		1.03±0.10		1.06±0.16		1.06±0.14		1.00±0.19		1.06±0.13		1.09±0.15		1.05±0.15		1.07±0.15		1.05±0.15		1.05±0.17		1.04±0.15	
	1 min hold	0.92±0.12		0.91±0.17		0.92±0.17		0.93±0.10		0.95±0.09		0.97±0.14		0.99±0.19		0.97±0.16		0.95±0.11		0.89±0.19		0.91±0.12		0.91±0.13	
	3 min hold	1.08±0.22		1.14±0.37		1.13±0.39		1.15±0.43		1.13±042		1.11±0.39		1.12±0.37		1.13±0.36		1.13±0.40		1.10±0.39		1.12±0.38		1.10±0.35	
	5 min hold	1.03±0.15		0.99±0.13		1.05±0.19		1.07±0.19		1.03±0.15		1.04±0.15		1.02±0.15		1.03±0.16		1.00±0.15		1.02±0.15		1.00±0.16		1.02±0.17	
	7 min hold	1.03±0.09		1.01±0.08		0.96±0.10		0.98±0.10		0.99±0.12		1.00±0.10		0.99±0.11		0.97±0.09		0.96±0.10		0.98±0.10		0.98±0.10		0.97±0.08	
100 mmHg	20 sec hold	1.06±0.13		1.02±0.08		1.09±0.15		1.05±0.14		1.05±0.14		1.07±0.21		1.02±0.13		1.00±0.14		0.98±0.14		1.04±0.11		1.11±0.20		1.13±0.18	
	1 min hold	0.98±0.13		1.00±0.12		1.02±0.15		1.01±0.13		1.00±0.14		1.04±0.12		0.99±0.16		0.97±0.12		0.96±0.14		0.99±0.14		0.99±0.13		1.00±0.13	
	3 min hold	1.04±0.27		1.02±0.25		1.02±0.48		1.08±0.53		1.06±0.48		1.07±0.42		1.06±0.36		1.06±0.36		1.06±0.42		1.04±0.141		1.04±0.40		1.03±0.33	
	5 min hold	0.97±0.10		1.03±0.18		0.99±0.10		0.96±0.07		0.97±0.12		0.98±0.08		0.96±0.07		0.95±0.07		0.95±0.07		0.94±0.06		0.96±0.09		0.97±0.11	
	7 min hold	1.03±0.16		1.11±0.13		1.14±0.28	ψ	1.12±0.24	ψ	1.10±0.23		1.09±0.21		1.08±0.20		1.06±0.15		1.07±0.19		1.04±0.21		1.08±0.17		1.13±0.20	
200 mmHg	20 sec hold	1.07±0.14		1.09±0.14		1.16±0.17		1.16±0.19		1.17±0.19		1.15±0.18		1.13±0.19		1.14±0.20		1.16±0.16		1.15±0.13		1.16±0.18		1.16±0.22	
	1 min hold	1.11±0.14		1.09±0.11		1.16±0.14		1.13±0.16		1.12±0.17		1.12±0.17		1.12±0.17		1.10±0.16		1.11±0.16		1.10±0.16		1.10±0.15		1.09±0.15	
	3 min hold	1.14±0.16		1.15±0.17		1.18±0.16	ψ	1.22±0.13	ψ	1.23±0.15	ψ	1.19±0.17	ψ	1.20±0.17	ψ	1.19±0.18	ψ	1.21±0.16	ψ	1.21±0.17	ψ	1.19±0.18	ψ	1.20±0.17	ψ
	5 min hold	1.02±0.10		1.09±0.11		1.08±0.12	ψ	1.10±0.12	ψ	1.09±0.11	ψ	1.08±0.12	ψ	1.08±0.11	ψ	1.09±0.12	ψ	1.08±0.12	ψ	1.07±0.13	ψ	1.07±0.11	ψ	1.06±0.10	ψ
	7 min hold	1.06±0.14		1.09±0.13		1.14±0.14	ψ	1.16±0.14	ψ	1.14±0.15	ψ	1.15±0.13	ψ	1.16±0.15	ψ	1.14±0.14	ψ	1.13±0.15	ψ	1.14±0.14	ψ	1.12±0.14	ψ	1.13±0.14	ψ

## Discussion

In the analysis of the StO_2_ values by cuff pressure, no increase in StO_2_ was observed after release at 50 mmHg compared to that in the resting state; however, several points where StO_2_ values increased at higher cuff pressures of 100 and 200 mmHg were observed. At 100 mmHg, the longer the holding time, the lower the StO_2_ value at the time point just before release, and the duration of the StO_2_ increase became longer in response. 

The same trend was observed at 200 mmHg; however, the duration of the high StO_2_ value was longer. Analysis of StO_2_ values at the time point immediately before release revealed that higher cuff pressures (50, 100, and 200 mmHg) and longer holding times yielded lower values. The fact that no increase in the value occurred after release at 50 mmHg suggests that the value at the time point immediately before release did not reach the threshold necessary to exert a vasodilatory effect. From these observations, it is evident that a cuff pressure of 100 mmHg or higher is necessary to obtain a blood flow-promoting effect under the conditions of this study, and the longer the holding time at a cuff pressure of 100 mmHg or higher, the longer the blood flow-promoting effect. Analyzing the table of the total Hb, no change was observed at 50 mmHg; however, at 100 mmHg, the total Hb increased until two minutes after release with a holding time of seven minutes. At 200 mmHg, the total Hb level increased up to 10 minutes after release at holding times of three, five, and seven minutes. This indicates that with a higher cuff pressure and longer holding time, the total Hb level was higher. The increase in the total Hb levels suggests an increase in blood content. A decrease in StO_2_ and an increase in total Hb can be suggestive of congestion. However, in this case, StO_2_ was elevated; therefore, it was not considered congested. A possible cause of the increase in the total Hb level is the relaxation of the vascular smooth muscle. The concentration of CO_2_ produced by metabolism in the muscle cells increases when a state of restricted blood flow is maintained. Carbon dioxide relaxes the vascular smooth muscle cells. Hydrogen ions generated by CO_2_ hydration have been suggested to inhibit Ca^+^ channel activity and reduce contractility by decreasing the intracellular pH in the vascular smooth muscle [11-14].

In an organism, blood flow to the capillary network is determined by the vascular smooth muscle (pre-capillary sphincter) of the arterioles in front of it, and this vascular smooth muscle is controlled by the sympathetic vasoconstrictor nerve. Normally, not all anterior capillary sphincters remain open. Some are temporally closed, and a network of dormant capillaries is present. Owing to the relationship between the degree of blood flow restriction and retention time, the CO_2_ concentration may have increased sufficiently to relax multiple vascular smooth muscle cells allowing blood flow in the dormant capillary network, leading to an increase in the blood content of the muscle. This experiment demonstrated that the promotion of muscle blood flow depends on two factors: pressure and holding time. An increase in StO_2_ and blood circulation to the resting capillary network for two minutes was observed under a pressure of 100 mmHg and a holding time of seven minutes, suggesting enhanced blood flow. Similarly, an increase in StO_2_ and blood circulation to the resting capillary network for at least 10 minutes was observed under the conditions of 200 mmHg pressure and holding times of three, five, and seven minutes, suggesting enhanced blood flow. Therefore, these conditions seem to be appropriate for flushing pain- and fatigue-related substances from the muscles.

Perspectives and significance

In this study, the cuff air pressure and holding time necessary to increase muscle blood flow were determined. In the future, we will perform a squeeze-hold test under these conditions in patients with myogenic pain to further verify its effectiveness. Specifically, we will consider adaptations for delayed-onset muscle soreness (DOMS) and muscle fatigue. DOMS refers to the muscle pain that occurs the day after or the day following excessive exercise. Muscle fatigue results from the accumulation of fatigue-inducing substances in the muscles, leading to a decline in muscle performance. Both DOMS and muscle fatigue can significantly restrict daily activities, exercise habits, and athletic performance. However, effective means of alleviating DOMS and muscle fatigue have not yet been reported. Consequently, there is considerable interest in the rehabilitation and sports fields regarding the mitigation of these conditions. The enhanced muscle blood flow achieved through the squeeze-hold technique is expected to maximally flush out pain-related substances and fatigue-inducing compounds from the muscles, potentially demonstrating significant effectiveness in alleviating DOMS and muscle fatigue. If an effective method for alleviating DOMS and muscle fatigue is established, its contributions to the fields of rehabilitation and sports could be immeasurable.

Limitations of the study

The limitations of this study include low sample size, no control group, and absence of long-term data. Moreover, the findings are limited to young healthy male volunteers and cannot be directly generalized to women and elderly individuals with low muscle mass or patients with clinical conditions affecting blood flow. For clinical application, further study is necessary.

## Conclusions

This study elucidates the effects of air pressure and holding time on muscle blood flow promotion using a pneumatic squeeze-hold technique. The results indicate that a cuff pressure of at least 100 mmHg is required to achieve a significant increase in muscle blood flow, with longer holding times at higher pressures resulting in more sustained blood flow enhancement. Specifically, at 100 mmHg, the duration of increased blood flow extended up to eight minutes, while at 200 mmHg, the effect persisted for up to 10 minutes post-release. These findings highlight the potential of the pneumatic squeeze-hold method in enhancing muscle blood flow, suggesting its application in clinical settings for the management of muscle-related pain and fatigue. The limitations of this study include a small sample size, the absence of a control group, and the lack of long-term data. Future studies should explore its efficacy in diverse populations, including elderly individuals with lower muscle mass and patients with clinical conditions affecting blood flow.

## References

[1] Nakajima M, Tsuro T, Endo A (2022). Sustained compression with a pneumatic cuff on skeletal muscles promotes muscle blood flow and relieves muscle stiffness. Int J Environ Res Public Health.

[2] Green DJ, Dawson EA, Groenewoud HM, Jones H, Thijssen DH (2014). Is flow-mediated dilation nitric oxide mediated?: A meta-analysis. Hypertension.

[3] Ghimire K, Zaric J, Alday-Parejo B (2019). MAGI1 mediates eNOS activation and NO production in endothelial cells in response to fluid shear stress. Cells.

[4] Ignarro LJ, Buga GM, Wood KS, Byrns RE, Chaudhuri G (1987). Endothelium-derived relaxing factor produced and released from artery and vein is nitric oxide. Proc Natl Acad Sci U S A.

[5] Mendoza SA, Fang J, Gutterman DD (2010). TRPV4-mediated endothelial Ca2+ influx and vasodilation in response to shear stress. Am J Physiol Heart Circ Physiol.

[6] Hall J, Guyton A (2006). Textbook of Medical Physiology (11th ed). Textbook of Medical Physiology (11th ed). Philadelphia, USA: ELSEVIER.

[7] McCully KK, Hamaoka T (2000). Near-infrared spectroscopy: what can it tell us about oxygen saturation in skeletal muscle?. Exerc Sport Sci Rev.

[8] De Blasi RA, Ferrari M, Natali A, Conti G, Mega A, Gasparetto A (1994). Noninvasive measurement of forearm blood flow and oxygen consumption by near-infrared spectroscopy. J Appl Physiol (1985).

[9] Homma S, Eda H, Ogasawara S, Kagaya A (1996). Near-infrared estimation of O2 supply and consumption in forearm muscles working at varying intensity. J Appl Physiol.

[10] Van Beekvelt MC, Colier WN, Wevers RA, Van Engelen BG (2001). Performance of near-infrared spectroscopy in measuring local O(2) consumption and blood flow in skeletal muscle. J Appl Physiol (1985).

[11] Zochodne DW, Ho LT (1993). Evidence that capsaicin hyperaemia of rat sciatic vasa nervorum is local, opiate-sensitive and involves mast cells. J Physiol.

[12] Austin C, Wray S (1995). The effects of extracellular pH and calcium change on force and intracellular calcium in rat vascular smooth muscle. J Physiol.

[13] Klöckner U, Isenberg G (1994). Intracellular pH modulates the availability of vascular L-type Ca2+ channels. J Gen Physiol.

[14] Klöckner U, Isenberg G (1994). Calcium channel current of vascular smooth muscle cells: extracellular protons modulate gating and single channel conductance. J Gen Physiol.

